# Common and Trace Metals in Alzheimer’s and Parkinson’s Diseases

**DOI:** 10.3390/ijms242115721

**Published:** 2023-10-29

**Authors:** Julia Doroszkiewicz, Jakub Ali Farhan, Jan Mroczko, Izabela Winkel, Maciej Perkowski, Barbara Mroczko

**Affiliations:** 1 Department of Neurodegeneration Diagnostics, Medical University of Bialystok, 15-269 Bialystok, Poland; 2 Department of Public International Law and European Law, Faculty of Law, University of Bialystok, 15-089 Bialystok, Poland; 3 Dementia Disorders Centre, Medical University of Wroclaw, 50-425 Scinawa, Poland

**Keywords:** neurodegeneration, Codex Alimentarius, heavy metals, trace elements, food, diet

## Abstract

Trace elements and metals play critical roles in the normal functioning of the central nervous system (CNS), and their dysregulation has been implicated in neurodegenerative disorders such as Alzheimer’s disease (AD) and Parkinson’s disease (PD). In a healthy CNS, zinc, copper, iron, and manganese play vital roles as enzyme cofactors, supporting neurotransmission, cellular metabolism, and antioxidant defense. Imbalances in these trace elements can lead to oxidative stress, protein aggregation, and mitochondrial dysfunction, thereby contributing to neurodegeneration. In AD, copper and zinc imbalances are associated with amyloid-beta and tau pathology, impacting cognitive function. PD involves the disruption of iron and manganese levels, leading to oxidative damage and neuronal loss. Toxic metals, like lead and cadmium, impair synaptic transmission and exacerbate neuroinflammation, impacting CNS health. The role of aluminum in AD neurofibrillary tangle formation has also been noted. Understanding the roles of these elements in CNS health and disease might offer potential therapeutic targets for neurodegenerative disorders. The Codex Alimentarius standards concerning the mentioned metals in foods may be one of the key legal contributions to safeguarding public health. Further research is needed to fully comprehend these complex mechanisms and develop effective interventions.

## 1. Introduction

A set of brain illnesses known as neurodegenerative diseases have a clinical history that is progressive and frequently incurable. The majority of cases have a multifactorial etiology due to interactions between genotype, lifestyle, and environmental variables; genetic alterations have a recognized causal role in these instances. Because a greater proportion of the population is exposed to industrial and chemical pollution through food, air, and water, metals are receiving more attention as environmental risk factors [1]. Not only is neurodegeneration linked to unknown etiology, but also other neurological diseases, such as autism, or mental health issues like depression. Many physiological processes depend on different metals, and their homeostasis is rigorously managed since their buildup or lack can cause different illnesses in humans. Since the precise process by which metals cause toxicity is still not fully known, it is conceivable that various metals might cause toxicity in different ways. Particularly significant are the effects of hazardous exposures to critical metals on oxidative stress, neurodegeneration, and dyshomeostasis in essential metal metabolism [2]. The glutathione system, thioredoxin/peroxiredoxin system, superoxide dismutases, and catalase are a few of the processes that protect brain cells from oxidative stress. Cell death results from an imbalance brought on by higher amounts of oxidant substances or lower levels of the antioxidant systems. Metals may cause DNA damage and trigger apoptosis through the generation of reactive oxygen species (ROS) and interactions with cell signaling pathways [3]. The brain is particularly vulnerable to oxidative stress, which can be made significantly harsher by the presence of redox-active metals due to their high oxidative metabolic activity and comparatively weak antioxidant defense. Epigenetic factors may potentially contribute to the development of neurodegenerative disorders. DNA methylation, DNA methylation-bound histones, and chromatin remodeling may all be influenced by environmental cues, including a person’s lifestyle, food, and exposure to toxins. The induction of genes linked to neurodegenerative illnesses may also be increased by the effects of metals on gene expression [4,5]. 

## 2. Methods

**Figure 1:**
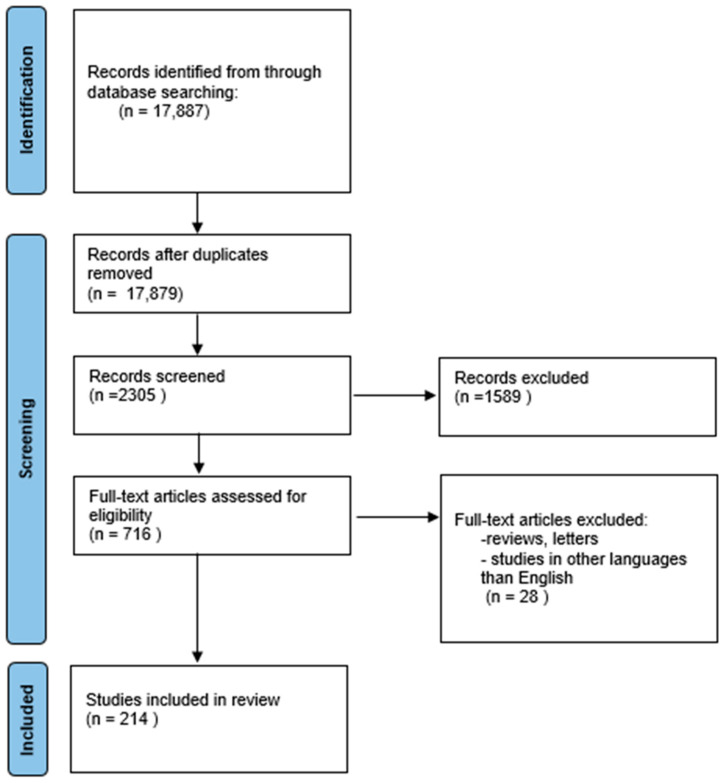
Prisma flow diagram depicting the methods for including studies in the review.

We performed a comprehensive literature search covering the period up to 26 July 2023. First, we searched the MEDLINE/PubMed electronic database using the keywords “metals AND neurodegenerative diseases” (n = 10,870) and “heavy metals AND neurodegenerative diseases” (n = 7017). The next step involved choosing metals important for the review. Additionally, we used keywords related to neurodegeneration: “Alzheimer AND metals” (n = 7327) and “Parkinson AND metals” (n = 4278). The next step involved limiting the search to studies written in English and the exclusion of duplicates. Thus, 214 publications were included in the study (Figure 1, PRISMA flow diagram modified from Page et al. [6]).

## 3. Essential Metals to the Healthy Brain

### 3.1. Zinc

Zinc (Zn) is a transition metal that is essential for humans and numerous other organisms. It is the second most common transition metal after iron. The duodenum and jejunum of the small intestine are where dietary Zn is absorbed before being transported to nearby tissues. The excess zinc is eliminated by mucosal cell sloughing, gastrointestinal discharge, and tegument [7,8]. The metal affects the immune system and has neuroprotective qualities, and fluctuations in plasma levels have effects on the immunological and central nervous systems in particular [7,9]. Moreover, Zn is an essential metal for convenient brain function. It takes part in catalytic and protein structure-stabilizing processes, and its deficiencies are associated with several detrimental disorders, such as immune system impairment, reduced skin and bone development and repair, and cognitive dysfunction [10]. The significance of Zn in CNS function is being increasingly recognized, and it has been proposed that it has a major role in the emergence of various NDDs, including AD, PD, and MS [9,11]. Zn is present in the CNS in two different forms: first, it is tightly linked to proteins, and second, it is present in presynaptic vesicles in its free, cytoplasmic, or extracellular form. Both ionotropic and metabotropic postsynaptic receptors are modulated by Zn released from synaptic vesicles under normal circumstances. There are a variety of unique ways to transport Zn across the cellular membrane that are necessary for Zn to carry out its many bioactive activities [12]. 

In many various regions of the brain, including the cerebral cortex, hippocampus, amygdala, and auditory brainstem, zinc is localized in the synaptic vesicles of vast populations of glutamatergic neurons. This synaptic zinc pool, which is labile and targets a wide range of postsynaptic receptors, is released in an activity-dependent way [10,13]. The negative effects on NDDs provide further evidence of the physiological significance of Zn homeostasis in humans. As an example, excessive Zn inhibits the absorption of Cu and Fe, promotes the generation of ROS in the mitochondria, interferes with the function of metabolic enzymes, and triggers apoptotic processes [14]. Numerous inflammatory cytokines can produce and signal differently depending on the type of cell, and Zn can affect this process. During the acute phase of a reaction to various stimuli, such as stress, illness, and trauma, plasma Zn concentrations rapidly decrease. As a result, Zn is transported into various cellular compartments where it is used for protein synthesis, free radical neutralization, and microbial invasion prevention. During inflammatory processes, Zn seems to be redistributed, and cytokines appear to be the mediators. Therefore, hypozincemia and increased cytokine production are both present in individuals with acute diseases [7,15]. Additionally, Zn consumption may affect cytokine production in people with chronic inflammation, which is defined by elevated levels of inflammatory cytokine production. Compared to individuals with higher Zn consumption, those with lower Zn dietary intake have increased gene expression of inflammatory cytokines such as interleukin (IL)-1α, IL-1β, and IL-6 and lower plasma and intracellular Zn concentrations [16]. Additionally, Zn influences key pro-inflammatory signaling pathways as an anti-inflammatory element. Zn stops nuclear factor kB (NF-kB) from separating from the inhibitory protein that it is paired with, preventing NF-kB from translocating into the nucleus and suppressing subsequent inflammation. Zn also prevents STAT3 from being activated by IL-6 [15].

### 3.2. Copper

Copper is another essential metal for living organisms. It is the third-most prevalent necessary transition metal in the human liver, after iron and zinc. In the brain, it is essential for preserving the redox equilibrium [17]. Despite the fact that the brain has a high need for metals, copper (Cu) is a crucial trace element for people and an important cofactor in electron transfer processes [18]. Additionally, the buildup of metal elements in tissues is linked to aging or age-related illnesses, including cancer, neurological diseases like Alzheimer’s and Parkinson’s, and metabolic diseases like diabetes. Cu is absorbed through the digestive system, stored in the liver, and mobilized into circulation; however, it is unclear how Cu is maintained throughout the body as a whole [19]. 

Cu may enter the brain in a regulated way through the Cu transporter, which is situated at the brain barriers and is essential for neuronal growth, maturation, and functioning. Copper homeostasis in the brain is regulated via the blood-brain barrier (BBB) and blood-cerebrospinal fluid barrier (BCB) [19,20]. The blood-brain barrier was shown to be a primary entry point for Cu into the parenchyma of a rat brain. Particularly, it was discovered that the blood-CSF barrier regulates brain Cu homeostasis. The extra copper is released from the brain cells into the cerebrospinal fluid (CSF) if there is an excess of copper, where it is subsequently taken up by the cells that make up the blood-cerebrospinal fluid barrier. These cells either retain the copper they absorb in anticipation of transfer to the CSF by ATP7B or release it into circulation by ATP7A. The brain has the third-highest concentration of copper of any organ, making up around 9% of the body’s copper [19,21]. The brain region with the highest concentration of copper, known as the locus coeruleus (LC), is principally responsible for creating the neurotransmitter norepinephrine (NE), which is produced by brainstem neurons [17]. Through the LC, copper alters rest-activity cycles. The BBB and BCB barriers maintain a careful equilibrium of copper in the central environment [20]. Glial cells have a larger quantity of copper than neurons do, both in healthy and unhealthy situations, according to histochemistry experiments carried out on brain slices [19,22]. Regardless of the source of stimulation or the cell’s origin, senescent cells collect intracellular copper, and this is probably a universal phenomenon [23]. 

In order to absorb dietary cupric Cu (Cu^2+^) across the apical membrane, Cu transporter 1 (CTR1), a high-affinity Cu uptake transporter, must convert it to cuprous Cu (Cu^1+^). Numerous reductases, including ferrireductase, duodenal cytochrome, or cytochrome b reductase 1 (DCYB), and the six-transmembrane epithelial antigen of the prostate-2 (STEAP2) metalloreductase, are considered to be involved in the reduction. In the intestinal epithelial cells, CTR1, which localizes to the apical membrane and early endosomes, Cu^1+^ is taken up. The liver is where Cu is loaded onto ceruloplasmin for systemic circulation after it binds to albumin or 2-macroglobulin in the blood and is delivered there from the intestinal epithelial cells. While the latter is required for Cu mobilization into the mitochondria, the former is useful for loading Cu to SOD1. The metallothioneins attach to extra Cu in the cytosol, lowering the amount of free Cu ions, which is considered to be crucial for preventing the toxicity brought on by free Cu ions. Cu is weakly coupled to albumin; therefore, it is feasible that at the BBB, Cu ions might be liberated from the albumin-bound moiety and then transported into the brain [7,24]. 

### 3.3. Iron

Many biological activities in the brain, including DNA synthesis, oxygen transport, myelination, producing neurotransmitters, and mitochondrial functioning, depend on iron (Fe). For the brain to function normally in terms of physiologically, iron homeostasis must be preserved. The homeostatic system gives cells the best chance to perform a number of critical tasks, such as maintaining the equilibrium state of iron concentrations inside the cells and buffering chemicals to prevent harmful accumulation inside cellular compartments. The concentration of iron can rise when the iron level exceeds the capability for iron sequestration. This can result in many cellular organelles being damaged and brain cell death. The regular functioning of the cells may be hampered by both greater iron deposition or iron shortage [25]. Fe is absent from the brain at birth; it quickly increases between youth and middle age and then stays largely constant [26,27,28]. Age-related Fe buildup mostly affects basal ganglia and other brain areas related to motor skills [29,30]. The red nucleus, substantia nigra (SN), and putamen exhibit the sharpest increase during adult life [26]. The substantia nigra pars compacta (SN), circumventricular organs, globus pallidus, and oligodendrocytes are where iron is mostly distributed in the brain [25]. Therefore, interest in understanding iron metabolism in the CNS is growing. The brain’s iron absorption is mediated by endothelial TfR1. However, the amount of iron present in the CNS controls how much TfR is expressed. In addition, the GPI-anchored melanotransferrin, lactoferrin, and lactoferrin receptor (LfR) all play crucial roles in iron transport across the BBB. The BBB can also contain iron that is not linked to transferrin (NTBI) [25].

The Tf/TfR system, which may be the main mechanism for iron transport through the luminal membrane of the endothelium, is where our current understanding of iron homeostasis in the brain starts. Iron that is linked to transferrin is subsequently transformed into Fe^3+^ and moved through the endosomal membrane by DMT1. Tf will re-enter the luminal membrane after TfR releases iron. Other mechanisms, such as GPI-anchored melanotransferrin/soluble melanotransferrin and lactoferrin receptor/lactoferrin, may also be implicated in the transport of iron across the BBB. In addition to these routes, transcytosis is another method by which transferrin-bound iron crosses the BBB. Iron may be transported across the BBB as Fe^2+^ via FPN1 and hephaestin. Astrocytes may also be involved in the transport of iron across the BBB [25]. On the basolateral surface of endothelial cells, FPN1 releases Fe^2+^. Following that, astrocytes oxidize Fe^2+^ into Fe^3+^ by CP through their end-feet processes on the capillary endothelia, and these products are further bound with Tf produced by the choroid plexus and oligodendrocytes. This suggests that iron enters the body not only through the BBB’s endothelial cells but also through the epithelial cells of the choroid plexus. Iron will diffuse via CSF and the interstitial fluid of the brain parenchyma and bind with Tf after iron enters into interstitial fluid or CSF in ventricles. This will deliver iron to the cells expressing TfR inside the CNS [25,31,32]. 

### 3.4. Manganese

The growth of the nervous system, the brain, and cognitive function all depend on manganese. For the majority of adults, food intake typically meets the necessary Mn intake. Arginase, agmatinase, glutamine synthetase, and Mn superoxide dismutase (MnSOD) are just a few of the Mn-dependent enzymes. These enzymes all serve special roles that Mn plays that cannot be substituted by other metals. Because of this, it must be present in the body at appropriate levels to support these processes, and its absence may result in cognitive deficiencies. Excessive cellular Mn levels are also harmful, and the discipline of toxicology has begun to pay more attention to this component of Mn-induced illness. Mn builds up in particular parts of the brain, changing neurophysiology just in those areas. Elevated brain Mn levels often only happen after overexposure, which can be caused by a variety of things, including environmental factors, work-related exposures, or dietary sources of Mn, including tainted water [33]. The major ways to be exposed to Mn are through eating, skin absorption, and inhalation. In addition, whole grains, nuts, seeds, tea, legumes, pineapple, and beans are the main sources of Mn in the diet. The gut is where ingested Mn is absorbed; however, its molecular processes of absorption are poorly understood. Divalent metal transporter 1 (DMT-1) and passive diffusion are considered to be the two ways that Mn might enter cells [7]. 

**Figure 2:**
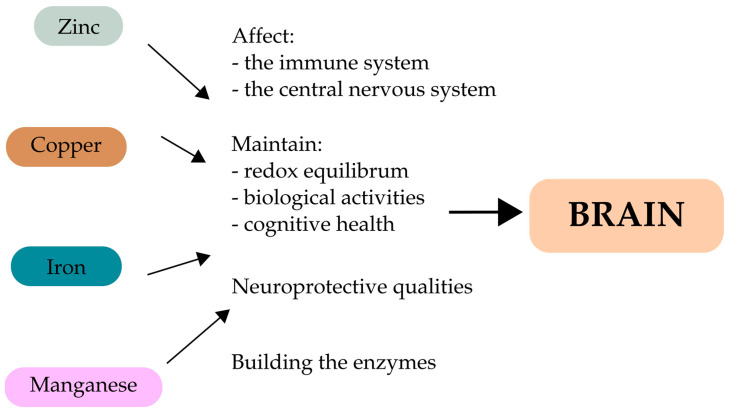
Beneficial effects of Zn, Cu, Fe, and Mn on a healthy brain.

Several enzyme families, including oxidoreductases, transferases, hydrolases, lyases, isomerases, and ligases, depend on manganese (Mn), an important trace element. As a result, Mn plays a role in the metabolism of amino acids, lipids, proteins, and carbohydrates, as well as protein glycosylation, immune function, blood sugar regulation, cell energy production, reproduction, digestion, bone growth, and blood clotting [34,35,36]. The metalloenzyme Mn superoxide dismutase, which is found in the mitochondria and aids in the detoxification of superoxide free radicals, is another structure that Mn takes on. The main source of Mn, which is omnipresent in many foods, is our diet. Grains, leafy greens, fruits, nuts, spices, and tea are all particularly high in Mn [34,35,36]. Only 1% to 5% of ingested Mn is absorbed from the colon, meaning that the gut closely regulates the body’s Mn burden [36]. The liver, the primary regulator of Mn disposal, immediately excretes any extra Mn present in the portal circulation into the bile after Mn absorption. Enterohepatic circulation affects a sizeable portion of biliary excreted Mn [35]. A higher intake of Mn through food causes homeostatic adaptation, which decreases gastrointestinal Mn absorption and increases biliary Mn excretion. A number of dietary circumstances, including the availability of other trace elements, can also impact how much Mn is absorbed [37]. Its interaction with iron (Fe) has received a lot of attention. High Fe consumption lowers blood Mn levels, whereas anemia due to Fe shortage increases intestinal Mn absorption. The absence of nutritional Mn shortage in humans has been linked to the mineral’s pervasiveness in food. However, research on Mn shortage has shown that it might result in impaired glucose tolerance, ataxia, poor bone development, and skeletal deformities. Divalent metal transporter 1 (DMT1) is hypothesized to be involved in the absorption of Mn into the enterocyte in the proximal small intestine. DMT1 transports a variety of cations, including Mn, Fe, and others. DMT1 is an H^+^ symporter that moves one H^+^ and one divalent cation in the same way. All tissues contain DMT1; however, the duodenum has the highest concentrations [36,38]. What is more, DMT1 expression is influenced by Fe status, with enhanced DMT1 expression at the enterocyte membrane during Fe deficit [39]. As a result, Mn absorption through the colon increases with Fe shortage, leading to Mn buildup in the brain. On the other hand, diets rich in Mn result in lower plasma levels of Fe while raising levels of transferrin (Tf) and total iron-binding capacity (TIBC) [36]. Figure 2 displays the positive effects of essential metals on brain health. 

Interestingly, despite playing a crucial part in several metabolic processes, high Mn exposure has been linked to basal ganglia system failure, which results in a severe neurological illness akin to Parkinson’s disease (PD). Regardless of having pathologic and clinical differences, Mn-induced parkinsonism and Parkinson’s disease share generalized bradykinesia, widespread rigidity, and pathophysiological mechanisms that are largely the same, such as oxidative stress, protein aggregation, impaired proteasomal and autophagy functions, excitotoxicity, aberrant signal transduction, mitochondrial dysfunction, and cell death pathways [40]. The brain and the lungs, which are both essential organs, are both impacted by a Mn overload [41]. The shuffling gait of PD can be clearly distinguished from the dystonic high-stepping gait disruption linked to Mn toxicity [42]. 

## 4. Harmful Metals to the Brain

### 4.1. Lead

Throughout a person’s lifetime, lead is a toxin that is known to damage several bodily organs, notably the central nervous system [43]. Numerous studies have been carried out to determine how lead toxicity affects cognitive measures, and several meta-analyses have proven that exposure to even low levels of lead may be connected to lower intelligence, conduct issues, and symptoms of attention-deficit hyperactivity disorder like inattention and impulsivity [44,45,46]. In addition, several studies have found that modest levels of lead exposure may be linked to worse executive function, poorer language function, and worsened melancholy moods [47]. Basic neuroscientific research has identified a variety of processes through which lead may disturb brain circuits. Lead, for instance, acts as a replacement for calcium and improperly activates calmodulin-dependent processes, which, in turn, activate a variety of mechanisms that impair brain systems, including synapse formation, axon dendritic expansion, and plasticity. Lead also interferes with the release of neurotransmitters and the systems that control them, especially dopamine systems [48]. In particular, lead decreases stimulated neurotransmitter release while enhancing spontaneous neurotransmitter release [49]. Gamma-aminobutyric acid (GABA) release has been demonstrated to be reduced by lead, and the GABA pathway is also disrupted by lead [50]. Lead is absorbed by mitochondria, which results in swelling and functional disturbances. These effects cause cell death and turn excitotoxicity, which harms neurons, into normally innocuous synaptic transmission mediated by glutamate [43,51].

Lead neurotoxicity has a wide range of known repercussions on the central nervous system. Early lead exposure has been linked to lower blood-brain barrier integrity, altered myelination and synaptogenesis, increased iron deposition, and changes in brain metabolic composition, according to animal studies. The results from human magnetic resonance imaging studies support these findings, showing that lead exposure results in changed myelin microstructure, decreased structural brain volume, altered brain metabolite levels, and decreased activity in task-relevant brain circuits. Additionally, a small number of human imaging studies have connected MRI results to individual variations in cognitive function, indicating that brain alterations are probably a major factor in the neurobehavioral effects of lead exposure [52]. Moreover, lead exposure leads to the activation of enzymes that generate ROS in the brain. Including superoxide and hydroxyl radicals, they can cause oxidative damage to neuronal cells. They attack lipids, proteins, and DNA, leading to cell death and neuroinflammation. Chronic lead exposure is particularly harmful to children, as their developing brains are more susceptible to the pro-oxidant effects of lead. This can result in cognitive deficits, learning disabilities, and behavioral problems [53].

The main sources of Pb are Pb-based paints and Pb-contaminated dust from paint, and in earlier decades, leaded gasoline, and finally, outdated home plumbing or plants grown in polluted soil that may ingest Pb via their roots, creating two additional routes for Pb exposure through food and tap water [54]. Pb exposure-related neuropathology is characterized by a variety of effects, including but not limited to decreased cellular metabolism (particularly linked to mitochondrial dysfunction), abnormal protein utilization, messenger system dysfunction, cellular apoptosis, and altered regulation of gene transcription. Pb may traverse the blood-brain barrier because it can act as a replacement for calcium ions (Ca2); Pb does so with particular ease in growing brains. Due to its special tight junctions and other features, the BBB limits particle movement under normal circumstances. Moreover, Pb exposure has also been shown to harm the BBB. The characteristics of the endothelium of the microvessels that make up the BBB alter when Pb is added to the system. Due to endothelial cells’ attraction for Pb, tight junctions become dysfunctional, which results in the improper transit of molecules across the BBB [54,55]. Pb penetrates brain tissue through voltage-dependent Ca channels, where it reaches astroglia and neurons, among other cells. Researchers found that Pb exposure can harm glial cells, including oligodendroglia and astrocytes. Additionally, Pb has the ability to attach to, build up in, and damage mitochondria [54,56]. Cortical volume reductions can result from apoptosis, which has been linked to higher Pb exposure levels (as previously mentioned). Even a little exposure to Pb (<10 μg/dL) can result in cell death and apoptosis [54]. 

### 4.2. Cadmium

Cadmium is the naturally occurring bluish-white metal that is persistent in the environment and is found in the crust of the planet. The mining and refining industry, the burning of fossil fuels, the incineration and disposal of trash, and the creation and use of phosphate fertilizers are all examples of anthropogenic sources of cadmium [57]. The main source of cadmium exposure is diet, and smoking cigarettes is a significant source for both smokers and nonsmokers [58]. The major exposure pathways include eating contaminated food and breathing in cadmium-laden air. The International Agency for Research on Cancer has classed cadmium as a group I carcinogen and has determined that it serves no vital physiological purpose in humans [59]. Diabetes, osteoporosis, hypertension, decreased lung function, and kidney damage are all made more likely by prolonged exposure to low levels of cadmium [58]. Cadmium has recently been identified as a neurotoxin [60]. 

The gastrointestinal system and lungs are exposed to cadmium through consumption and inhalation. These tissues absorb cadmium, which then enters the circulation. Only trace levels of cadmium can normally get through the BBB in adulthood [61]. The blood-cerebrospinal fluid barrier is made up of the choroid plexus, which prevents blood toxins from entering the cerebrospinal fluid and preserves the internal, homeostatic milieu of the central nervous system. The primary location of cadmium buildup in the brain is the choroid plexus [62]. Cadmium may be transported directly to the brain through the olfactory nerve system, skipping the BBB in the process. With intranasal cadmium instillation in mice, cadmium concentrations in the olfactory mucosa and olfactory bulbs increased [63]. Odorant-evoked neurotransmitter release from the olfactory nerve and axonal projections from the olfactory epithelium to the olfactory bulbs were reduced when cadmium levels in the olfactory bulbs rose [64]. Mice exposed to cadmium performed worse on tests of olfactory memory and hippocampus-dependent spatial learning and memory [65]. Cadmium immediately enters the central nervous system through the olfactory system, where it inhibits adult neurogenesis in the hippocampus and olfactory bulb, resulting in long-lasting, irreparable damage [60]. Additionally, cadmium is another toxic metal that can have pro-oxidant effects on the brain. Cd exposure can stimulate ROS production through several mechanisms, including disrupting mitochondrial function and depleting antioxidants. Elevated ROS levels can lead to neurodegenerative diseases, such as Alzheimer’s and Parkinson’s, by contributing to protein misfolding and aggregation, inflammation, and neuronal damage [66].

Cadmium is taken up by cells through the divalent essential element transport mechanisms. Cadmium is transported through calcium, iron, and zinc transport systems (such as calcium channels, calcium transporter-1, and divalent metal transporter-1) [67]. Cadmium is predominantly absorbed through the digestive tract through DMT1, and this process is reliant on the body’s stocks of other metals, particularly iron. Through DMT1, iron insufficiency promotes intestinal cadmium absorption [68]. The brain’s neurons and vascular endothelial cells express DMT1, calcium transporters, and zinc transporters [60,69]. 

### 4.3. Aluminum

Aluminum has a long history of use in medicine as a low-side-effect treatment against pathological hyperhidrosis and as an adjuvant in vaccines [70]. The public discussion regarding aluminum’s propensity to cause cancer and its often very uncritical neurotoxic impact has, nevertheless, gained greater attention in recent years. Proteins have a strong affinity for aluminum (Al^3+^), which can cross-link them. The principal mechanism for brain aluminum absorption has been identified as aluminum crossing the blood-brain barrier. Carrier receptors are the main rate and extent mechanisms through which aluminum crosses the blood-brain barrier. One of the crucial carrier receptors that transport aluminum over the BBB is the Tf-TfR pathway. Aluminum may enter central nervous system cells through the same high-affinity receptor-ligand mechanism that has been proposed for iron transport to neurons and glial cells. Aluminum may enter the brain under physiologically normal circumstances, according to the definition. Tf belongs to a group of proteins that also includes serum Tf, ovotransferrin, and lactoferrin. These proteins bind to circulating Fe^3+^ to stop it from moving through the body in a hazardous state. The internalization of iron-loaded Tf results in the delivery and absorption of iron from Tf into cells, which is mediated by the TfR [71].

There is no known physiological purpose of Al in the human body, which is in contrast to other frequently occurring metals like iron, manganese, and zinc [72]. Dialysis patients have reported neurotoxic consequences that are clinically significant. The culprits were found to be aluminum salts, which had previously been introduced to the dialysate as a phosphate binder. Plasma and brain tissue samples from patients showed higher levels of aluminum [73]. Disorientation, memory issues, and dementia were seen in those afflicted [74]. First, the clearance of aluminum from the brain is slower than in other organs, which contributes to these consequences, and second, aluminum in the brain affects a wide range of biological effects [70,73]. Aluminum is able to alter hippocampal calcium signal pathways that are essential for neuronal plasticity and, consequently, for memory [75], in addition to generating oxidative stress and binding to negatively charged membrane structures in neurons. Aluminum neurotoxicity, which affects the production of the neurotransmitter acetylcholine [76], is particularly harmful to cholinergic neurons. The Alzheimer’s hypothesis, which postulates a link between aluminum and Alzheimer’s disease, specifically takes into account the latter two neurobiological consequences [72]. Once aluminum contamination was eliminated from the dialysate, aluminum-related neurotoxic effects may be partially reversed [70,74]. Aluminum is widely used in daily life and can be absorbed through various sources, including food, antacids, and cookware. In the brain, aluminum has been associated with the development of neurotoxicity. Al ions can promote ROS generation by activating microglia cells in the brain, leading to inflammation and oxidative stress. ROS-induced oxidative stress can result in damage to neural tissues, particularly in the hippocampus and the frontal cortex, affecting memory and cognitive function [77,78]. 

## 5. Heavy Metals in Neurodegenerative Diseases

### 5.1. Alzheimer’s Disease

Alzheimer’s disease is the most common progressive neurodegenerative disorder. Gross atrophy of the brain and hippocampus, amyloid-beta (Aβ) buildup into senile plaques, and hyperphosphorylated tau accumulation in neurofibrillary tangles are the hallmarks of neuropathology. The etiologies of AD are complex and include many variables. Familial forms consist of only about 5% of all cases of AD [79]. According to the postulated hypothesis, the brains of AD patients gradually accumulate Aβ, which is followed by the increasing deposition of Tau protein. Another theory contends that the most damaging elements influencing brain tissue are soluble oligomers of the Aβ and/or Tau proteins. Additionally, it was postulated that the immune system may have a role in the pathogenesis of AD. The immune system may identify the insoluble deposits of Aβ as foreign substances, which sets off a chain reaction of inflammatory reactions and damages neurons. The brain’s inflammation caused by amyloid plaques and NFTs is mostly due to the activation of microglia and astrocytes [80,81]. Throughout AD, synapses also lose their ability to function normally. Synapse loss is correlated with dementia, indicating that the degenerative process and the course of the illness depend on it. Neurites nearby bend and change course as a result of dense plaque deposition, which may alter how signals are sent at the synapses [82]. There is currently no cure for the disease, although much research has been conducted to find treatments to slow down the progression and also treat the disease. Therefore, efforts to include the various etiology routes and other pathogenic aspects of AD in a more comprehensive model are now required.

As previously described, metal ions play essential roles in the brain, and there is known evidence pointing to their homeostatic dysfunction across different neurodegenerative diseases, including AD. The “Metal Hypothesis” of Alzheimer’s disease and other neurodegenerative illnesses links neuropathology to the aging-related breakdown of metal ion homeostatic mechanisms. Therefore, even abnormal levels of essential trace elements might be harmful and contribute to the disease. Numerous pieces of research have linked increased zinc levels to plaque and neuropil disease; other studies have found lower zinc levels in the hippocampus and amygdala areas of AD patients. According to meta-analyses, AD patients’ blood zinc levels are considerably lower than those of healthy controls [83]. Numerous histochemical studies demonstrate zinc co-localization with plaque cores and blood vessels, as well as in the soma and dendrites of neurons with hyperphosphorylated tau. Age-matched, nondemented subjects do not show this trend. Zinc’s role in AD pathogenesis is supported by two primary lines of evidence: Zinc concentrations in the brain, blood serum, and cerebrospinal fluid are frequently AD biomarkers. The brain areas most afflicted by AD pathology have extensive innervation by zinc-containing axons, whereas those less impacted by pathology contain few zinc-containing terminals. Zinc flooding, which occurs when zinc-containing neurons produce sudden floods of free zinc into the extracellular spaces of the brain under a number of circumstances, such as traumatic brain injury, seizures, and ischemia, is a significant aspect of this examination [84,85]. Zinc attaches to Aβ and causes conformational changes that decrease the solubility of Aβ 1-42, which causes population shifts in polymorphic states, which right-shift the oligomer-aggregate equilibrium [86,87]. By producing conformational changes that lessen the likelihood of alpha-secretase cleavage, zinc cations (Zn^2+^) might affect APP processing, enhancing amyloidogenic APP cleavage. Enzymes such as disintegrin and metalloprotease 10 (ADAM10), which are triggered by zinc and catalyze non-amyloidogenic cleavage, complicate this pathway [86,87]. The aggregation of hyperphosphorylated tau is also associated with excess zinc. According to Wang et al., zinc can bind tau monomers directly and encourage conformations that are more likely to form oligomers and clusters [87]. Zinc has been shown in in vitro tests to be hazardous in that it may cause tubulin to aggregate, which leads to neurodegenerative morphological abnormalities, but only at extremely high, nonphysiological quantities [88]. According to Wang, zinc stimulates the Src-dependent pathways GSK-3, ERK1/2, JNK, and others that are thought to be involved in tau hyperphosphorylation. Zinc inhibits several proteins simultaneously, including protein phosphatase 2A (PP2A), which results in tau hyperphosphorylation and other metabolic abnormalities. Zinc chelators block Src, which, in turn, lessens the zinc-induced inhibitory phosphorylation of PP2A at Tyr307. This lessens tau hyperphosphorylation and aggregation in transgenic mouse models expressing human tau [85,87].

Cu interacts with amyloid precursor protein (APP) through a Cu-binding domain, which has a profound impact on the amyloid cascade [89]. Cu concentrations are elevated in Aβ and neurofibrillary tangles, where they also colocalize and control APP expression [90,91]. It has been hypothesized that the interaction of Aβ peptides with Cu and other metals may encourage gain-of-function processes that are essential for the emergence of Aβ neurotoxicity [92]. In this context, the modulation of reduction-oxidation (redox) cycles is the source of the Aβ-mediated “Cu toxicity”, which results in the formation of Aβ oligomers and their precipitation within plaques as well as lipid peroxidation through the production of H2O2 [93,94]. Small quantities of Cu consumed by drinking water double the plasma concentrations of nCp-Cu in preclinical AD models and also have an impact on the generation of Aβ [95]. Advanced glycation end-products (AGEs), which are harmful compounds seen in the AD brain and in diabetic patients, are also produced as a result of the collapse of Cu homeostasis [96]. By interacting with Cu to form hydroxyl radicals (OH), the free radical superoxide anions (O2) produced by the glycation of carbohydrates contribute to the oxidative stress associated with AD [97]. A loss-of-function theory of Cu-related toxicity has recently been put forth. The concept is predicated on the APP- Aβ complex’s poor capacity to manage Cu, which results in the extrusion of extra Cu across membranes [98]. There is strong evidence linking the variations in Cu levels in AD patients’ blood, brain, cerebrospinal fluid, and brain to the development of cognitive impairments and the passage between distinct phases of the AD-related spectrum [99]. 

The buildup of iron ions in the brain and the alteration in the expression of iron regulatory proteins in the iron metabolism pathway have been discovered to cause oxidative stress, which causes damage to the neuron spectrum [100,101]. According to numerous experimental findings, iron accumulation in AD patients’ brains is one of the causes of brain oxidative stress and is closely related to abnormalities in brain iron metabolism and some important iron homeostasis regulators, including ferritin protein, transferrin protein, FPN, etc. [100,102]. A lack of iron can also make it simpler for the body to absorb divalent elements, including lead (Pb), cadmium (Cd), aluminum (Al), and manganese (Mn). Even in the absence of excessive Mn in the environment or food, Fe deficiency can promote Mn buildup in the brain. The implications of Fe deficit on Mn transport for this event have been studied. Previous research has demonstrated that persons with Fe insufficiency had higher blood levels of Mn [7,103]. 

The most discussed metals considering AD are the heavy metals not found in a healthy brain. However, several studies have shed light on the role of Fe as an essential metal in healthy brains and Aβ aggregation and its implications on neurotoxicity. Liu et al. demonstrated that iron promotes the toxicity of Aβ peptides by interfering with their ordered aggregation. The cumulation of Aβ peptides is a key step in the formation of amyloid plaques, a hallmark of Alzheimer’s disease. These aggregates are believed to be neurotoxic and contribute to neuronal damage [104]. Iron binding to Aβ can disrupt the proper folding and assembly of these peptides, preventing their organized aggregation. The interaction of iron with Aβ peptides may lead to the formation of toxic intermediates. These intermediates could be structurally unstable and prone to generating reactive oxygen species, leading to oxidative stress and damage to neurons. As a result of iron’s interference with Aβ aggregation, there is an increased likelihood of neurotoxic species being present. These species may have a higher affinity for binding to neuronal cells, leading to the initiation of cytotoxic processes. Everett et al. further investigated the chemical speciation of β-amyloid/iron aggregates using soft X-ray spectro-microscopy. This advanced analytical technique allows researchers to examine the precise chemical environment of iron within Aβ aggregates [105]. Understanding the chemical speciation of iron in these aggregates is crucial for unraveling the mechanisms by which iron influences Aβ toxicity.

Numerous studies have shown that Aβ buildup, tau pathology, and inflammation—the hallmarks of AD—are influenced by either developmental or acute lead exposure. Early lead exposure in young rats raised APP and β-secretase 1 (BACE1) expression, which then caused Aβ buildup and plaque formation in the cortex and hippocampus, resulting in AD-like disease. Another investigation revealed that exposure to lead during the prenatal stage elevated the expression of APP and BACE1 in aged rat brains [106]. This is similar to how lead exposure during infancy elevated APP, BACE1, and transcription factor specific protein 1 (Sp1) expression and encouraged Aβ deposition in old monkeys [107]. Together, lead, arsenic, and cadmium exposure further improved the expression of APP and BACE1, which was followed by the greatest induction of A production [108]. Additionally, it has been demonstrated that developmental lead exposure activates the SREBP2-BACE1 pathway, disrupting the metabolism of cholesterol in the developing brain [109]. It is well established that the genesis of AD and the formation of Aβ are directly related to cholesterol dyshomeostasis in the brain [110]. Additionally, it has been demonstrated that acute lead exposure increases the buildup of Aβ in brain tissue and cerebrospinal fluid by impairing LRP-1-mediated clearance [111]. Notably, exposure to lead also raises the concentration of total and hyperphosphorylated tau. Lead exposure has been shown to raise tau and phosphorylated tau protein levels in SH-SY5Y neuroblastoma cells [112]. This is similar to how lead exposure during development raises the levels of CDK5, serine/threonine phosphatase activity, and tau protein, all of which help to produce NFTs later in life [113,114]. Inflammatory processes that cause neuronal death are also present in lead toxicity. People who have been exposed to lead have greater blood levels of TNF-α and granulocyte-colony-stimulating factors than those who have not [115]. Lead administration causes persistent glial activation, along with inflammatory and neurodegenerative characteristics, in a rat model [116]. The activation of microglia and the excessive production of proinflammatory proteins such as inducible nitric oxide synthase (iNOS), IL-1, and TNF-α have also been linked to lead exposure [117].

More notably, neurodegeneration is thought to be related to aluminum exposure [118]. There is a clear correlation between aluminum and AD, according to several studies that found a greater prevalence of AD or AD mortality in regions with high amounts of aluminum in drinking water [119]. Later research that showed aluminum’s capacity to cause neurofibrillary degeneration and encourage the emergence of tangle-like structures that matched the NFTs identified in the brains of AD patients validated this [120]. Additionally, NFT-containing neurons in AD brains were found to accumulate aluminum [121,122]. Higher tau aggregation, apoptosis, and neurological dysfunction were seen in animals that already had a pathological process causing tau aggregation but not in the controls when the effects of oral aluminum administration were studied using a tau mouse model showing slow progressive tau accumulation [123]. This suggests that aluminum has an aggravating effect on tau pathology. By increasing the activity of the tau kinases CDK5 and GSK-3, blocking tau dephosphorylation, and promoting tau aggregation, aluminum produces these effects [124,125]. It is interesting to note that glial cells preferentially take up aluminum, which causes them to produce inflammatory cytokines like IL-6 [32,126]. The CDK5/p35 cascade has been shown to be dysregulated by IL-6, which, in turn, has been shown to cause tau phosphorylation [127]. Aluminum therapy in rats has been associated with increased glial activation and inflammatory response [128], but more research is needed to determine if this glial activation is contributing to the acceleration of an early step in the development of AD pathogenesis. Aluminum was initially thought to have an impact on AD pathology through its interaction with tau, but it has now been shown that it also has an impact on Aβ by encouraging its synthesis, aggregation, and degradation [129]. Aluminum was given orally to AD mice, which caused an increase in the quantity of Aβ in both its secreted and accumulated forms, as well as an increase in plaque deposition [130]. Additionally, Aβ combined with aluminum is more hazardous than Aβ alone because it disrupts membranes and interferes with mitochondrial respiration and brain calcium homeostasis [131,132]. 

According to recent epidemiological investigations, the blood cadmium levels of older people were strongly linked to death from AD [133,134]. There is growing evidence that cadmium contributes to the formation of Aβ plaques in the AD brain [135,136]. The number and size of plaques increased in an in vivo investigation in which cadmium was added to the drinking water of APP/PS1 mice [135]. Cadmium ions may interact with the Aβ, which would then encourage plaque formation [137]. Additionally, it has been proposed that cadmium therapy inhibits the expression of neutral endopeptidase and β-secretase (ADAM10), both of which are crucial for lowering Aβ-levels in the brain [135,138]. The synergistic effects of cadmium, lead, and arsenic further promote amyloidogenic processing by raising the expression of APP, BACE1, and PSEN1, which raises the intriguing possibility that cadmium interacts with other metals in AD [108]. Cadmium affects Aβ but it also has a role in tau conformation and self-aggregation in the AD brain [139,140]. The third repetition (R3) of tau’s microtubule-binding domain has been shown to bind cadmium. As a result, the R3 domain adopts an α-helix shape that aids in tau self-aggregation and largely loses its random coil conformation. Additionally, cadmium therapy specifically inhibits muscarinic M1 receptors, which are known to adversely regulate GSK-3β and, therefore, increase total and phosphorylated tau protein [139,141,142]. These findings provide credence to the idea that cadmium may be contributing to the onset of AD [143]. The summary of results of described metals in AD patient’s brains is presented in Table 1. 

### 5.2. Parkinson’s Disease

**Figure 3:**
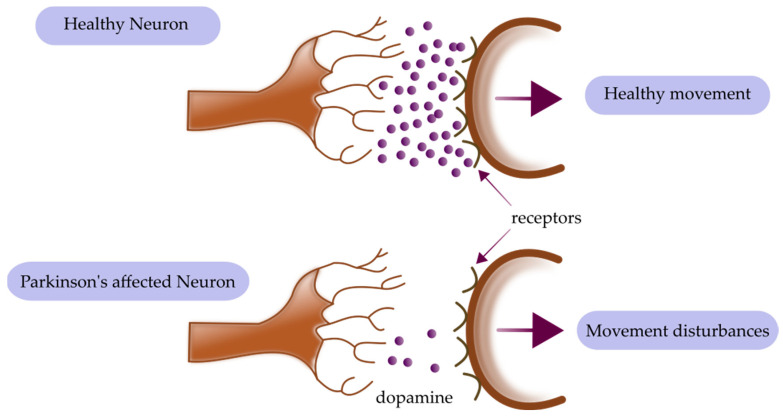
Schematic depiction of dopaminergic transmission changed in Parkinson’s disease.

Parkinson’s disease (PD) is the second most prevalent neurodegenerative condition [144]. The progression of PD is characterized by both motor and nonmotor symptoms, and it primarily affects adults over the age of 40, with the incidence rising with advancing age [145,146]. The most significant motor symptoms are tremors, bradykinesia, and postural instability, whereas the nonmotor symptoms include cognitive impairment, sleep difficulties, sadness, and anxiety [147]. The degeneration of dopaminergic neurons in the midbrain’s extrapyramidal pathway is one of the reasons thought to cause Parkinson’s disease and is thought to be the cause of motor dysfunction [148]. The scheme of dopaminergic transmission is presented in Figure 3. The presence of misfolded, insoluble α-synuclein, which may accumulate into Lewy bodies in neurons and cause neurodegeneration, is the histopathological sign of PD [149]. Similar to AD, neuroinflammation is associated with the pathophysiology of PD, where microgliosis and astrogliosis are observed [150]. Although the cause of PD is not fully understood, studies have shown that environmental variables predominate over genetic ones [151,152]. Heavy metals are naturally occurring substances that are among the environmental variables that contribute to the development of illness [153]. There are essential and non-essential sorts of these. Numerous proteins require the cofactors of metals, including manganese (Mn), copper (Cu), zinc (Zn), nickel (Ni), and iron (Fe). However, some heavy metals, such as cadmium, lead (Pb), and mercury (Hg), do not have biological effects and, instead, cause toxicity when consumed [154].

Age-related changes in the blood-brain barrier’s permeability, brain inflammation, changes in iron homeostasis, and other variables all contribute to an increase in total iron concentrations [155,156]. In the substantia nigra, globus pallidus, putamen, and caudate nucleus, iron concentrations rise with age [157,158]. It is unclear why this growth is specific to particular brain regions, though. In particular, iron builds up in the substantia nigra’s pars compacta and pars reticulata in connection with the severity of PD [159,160]. Studies using postmortem tissue have found that PD patients’ substantia nigras had significantly higher iron levels than people without the condition [161,162]. Iron is specifically elevated in the substantia nigra in different kinds of PD, which was shown by measurements made using transcranial sonography in vivo [163,164,165]. According to theory, the substantia nigra’s high iron concentrations, namely the toxic byproducts of iron-dopamine interactions, are what first cause neurotoxic effects [165,166]. Significant levels of iron have been shown to be neuromelanin-bound in substantia nigra neurons when using the X-ray fluorescence method. Therefore, iron that is coupled to neuromelanin may play a crucial role in protecting and storing this metal in neurons [167,168]. Experiments showing that iron chelation therapy has a neuroprotective effect support the idea that iron plays a pathogenic role in Parkinson’s disease (PD). Chelators like deferiprone, clioquinol, or deferoxamine are administered to PD animal models to treat motor impairments and stop the death of substantia nigra neurons [165,169,170,171]. 

Motor and sensory abnormalities, as well as neuropsychiatric and cognitive impairments, are symptoms of Mn poisoning [172,173]. The motor deficits include bradykinesia, “cock-gait”, fast postural tremor, hypertonia with cogwheel rigidity, and a propensity to trip when walking backward [174]. Neurons of the globus pallidus appear to be particularly susceptible to Mn-induced degeneration in both human patients and animal models [175], whereas the striatum is less severely impacted. In addition to the cerebellum, red nucleus, pons, cortex, thalamus, and anterior horn of the spinal cord, other brain regions may also be harmed by Mn poisoning [176]. This pathologic phenotype is different from idiopathic PD, which is characterized by a particular degeneration of dopaminergic neurons in the substantia nigra pars compacta [177]. Mn-induced parkinsonism can be distinguished from PD by the absence of Lewy bodies [177], the lack of therapeutic response to levodopa (a medication used to treat early stages of PD), the inability of PET studies to detect fluorodopa uptake, the occurrence of more dystonia, and the absence of resting tremor [178]. As previously described, αSyn is a protein that naturally aggregates into oligomers and is a major factor in the pathophysiology of PD. This is similar to how Mn^2+^ may cause the misfolding and buildup of the Syn protein, despite the fact that nuclear magnetic resonance (NMR) studies have demonstrated that Syn has a low affinity for Mn^2+^ in its C-terminal binding region [179,180]. Based on recent research, αSyn oligomerization is a primary cause of the neurotoxicity caused by Mn [181,182]. In a mouse model of Mn^2+^ exposure, Harischandra et al. showed that Mn also encourages the aggregation and prion-like exosomal transfer of Syn from cell to cell, leading to dopaminergic neurotoxicity [180,183]. These findings collectively suggest that Mn^2+^ exposure encourages Syn production in exosomal vesicles, which, in turn, triggers pro-inflammatory and neurodegenerative responses in both cell culture and animal models. Mn disrupts the cholinergic system, causing malfunction in the locomotor, affective, behavioral, and cognitive systems, according to numerous research. Mn has the ability to alter the activity of AChE and other cholinergic transmission-related enzymes [184,185]. AChE activity and cerebellar AChE expression were significantly increased in rats given Mn in drinking water for 30 days [184]. Similar to this, MnCl_2_ was temporarily administered to rats to increase AChE activity [186]. Additionally, brain extracts from rats fed a diet enriched in Mn displayed higher AChE activity compared to the control group in the study [185]. Due to the contradictory results following Mn exposure on AChE activity, Mn effects may vary depending on age, dose, route of exposure, frequency, and duration [180,185,187].

Mercury has been linked to an increased risk of Parkinson’s disease. A 1990–2008 study found a link between airborne mercury exposure and an increased incidence of Parkinson’s disease, especially in female nurses [188,189]. There are numerous possible causes of mercury exposure. China’s mercury mining, gold mining, and mercury-tainted food sources are some of these sources. The consumption of mercury-contaminated fish is the main cause of mercury exposure. Shark, tilefish, tuna, swordfish, king mackerel, pike, walleye, muskellunge, and bass are among the fish with a high mercury level. Devices that contain mercury, including thermometers, are sources of inorganic elemental mercury. When taking some laxatives or ingesting disc batteries, inorganic mercury salts might be present. Organic mercury can be found in contaminated seafood, mercury-based paints, thimerasol injections, and ingested products. Elemental mercury is typically exposed through inhalation or ingestion (inorganic mercury salts) [189,190]. Numerous brain functions, such as oxidative stress, decreased GSH levels, mitochondrial damage, and free radical aggregation, are impacted by mercury [191]. In the intestinal cell membrane, metalloproteins that would normally bind cuprous ions are rendered inactive by Hg because it prevents metalloproteins from chemically interacting with sulfur. As a result, free Cu causes toxicity, and the formation of Zn-Cu-SOD complexes is disrupted. Moreover, it causes Zn to be displaced in MT and SOD, which causes neurotoxicity [192]. Hg has also been linked to a rise in TNF-α, which promotes cellular death and neuroinflammation and results in symptoms similar to those of Parkinson’s disease. Patients with Hg exposure (such as those with dental amalgam fillings) are probably six times more likely to develop PD than nonexposed ones [193]. The summary of described metal effects on PD patients is presented in Table 2.

## 6. Dietary Approach to Neurodegenerative Diseases

Since there are no effective treatments for AD or PD, attention should be paid to prevention interventions such as dietary changes and other modifiable lifestyle variables. Numerous studies have shown that dietary habits and risk factors for neurodegenerative illnesses are closely related [194,195]. As an example, there is proof that the excessive consumption of saturated fat worsens neurodegeneration in AD and PD by boosting oxidative stress and lipid peroxidation [196,197,198]. What is more, researchers showed that higher saturated fat consumption causes an inflammatory response that attracts peripheral immune cells to the central nervous system, which may help to explain why the symptoms of the disorders described earlier increase [199]. Therefore, dietary modification has been studied as a potential treatment for the aforementioned disorders’ symptoms. This type of diet-based intervention is based on the idea that certain nutrients and metabolic substrates can have positive effects on neuroinflammation and neuronal function as well as on the disordered metabolic balance that is frequently observed in disorders of this nature. 

The most beneficial diet that was described for the prevention of neurodegenerative diseases is the Mediterranean diet (MD). Over time, the definition of the MD has evolved, although, in general, the MD is a diet high in fruits, vegetables, legumes, grains, nuts, fish, and monounsaturated fatty acids, low in dairy products, and somewhat low in red meats and dairy products [200]. The impacts of food items and their components with possible neuroprotective effects are credited for the health benefits of the Mediterranean diet. These goods include omega-3-rich fish and nuts, polyphenol-rich wine, and antioxidant-rich fruits, vegetables, and grains [201,202]. 

Chelators are compounds that have the ability to tightly bind to metal ions, effectively regulating their concentration in the body. In the context of AD and PD, chelators can play a significant role in mitigating metal ion dysregulation. This approach aims to restore metal ion homeostasis in the brain [157]. Chelators can be incorporated into dietary interventions to offer a more holistic and preventive strategy. Some dietary sources of natural chelators include flavonoids, polyphenols, and trace minerals like selenium and zinc. Additionally, some individuals may opt for chelator supplements under medical supervision [203]. The effectiveness of dietary chelators depends on their bioavailability and the ability to reach the brain in sufficient quantities. Responses to chelator-based dietary approaches may vary among individuals due to genetic factors, disease progression, and overall health status. It is crucial to consider the potential interactions between chelators and medications that individuals with AD and PD may be taking [204]. Research into the role of chelators in AD and PD is still evolving. Future studies may uncover more specific chelators with enhanced neuroprotective properties and improved bioavailability. Additionally, clinical trials will provide valuable insights into the practicality and efficacy of dietary chelator interventions [205].

## 7. Standards Regarding Metals in Food 

The issue presented above has a significant impact on public health. Diet is one of the environmental factors that affect Alzheimer’s and Parkinson’s diseases [206,207]. A variety of dietary elements that affect the incidence of Parkinson’s and Alzheimer’s disease have been shown by several in vitro, in vivo, and human epidemiological investigations [208,209]. It is the consumption of foodstuffs that is the most likely route for human exposure to heavy metals [210], as well as being the source of essential metals [211]. Therefore, it is not surprising that the complexity of the interaction of metals with human health, in the form of a dichotomous division into metals harmful to the brain and essential metals for a healthy brain, finds its reflection in food law. Food law related to this matter must be treated as an essential element of the strategy for preventing the development of the aforementioned neurodegenerative diseases. 

In the modern food trade, which is largely internationalized, and despite the fundamental differences in food laws among various legal systems, the aforementioned dichotomy can be observed based on regulations at even the universal level, such as the Codex Alimentarius. “The Codex Alimentarius is a collection of internationally adopted food standards and related texts presented in a uniform manner. These food standards and related texts aim at protecting consumers’ health and ensuring fair practices in the food trade. The publication of the Codex Alimentarius is intended to guide and promote the elaboration and establishment of definitions and requirements for foods to assist in their harmonization and in doing so to facilitate international trade” [212]. These food standards and related texts are developed by the Codex Alimentarius Commission, established by the Food and Agriculture Organization (FAO) and the World Health Organization (WHO). The Codex standards do not represent legally binding norms [213]. However, they have been referred to in the founding agreements of the World Trade Organization (WTO) and have become a reference point for national standards [214].

When it comes to heavy metals, which are treated as contaminants, the General Standard for Contaminants and Toxins in Food and Feed has set standards for the following metals: arsenic, cadmium, lead, mercury, methylmercury, and tin. A contaminant is “[a]ny substance not intentionally added to food or feed for food producing animals, which is present in such food or feed as a result of the production (including operations carried out in crop husbandry, animal husbandry and veterinary medicine), manufacture, processing, preparation, treatment, packing, packaging, transport or holding of such food or feed, or as a result of environmental contamination”. Regarding lead, maximum levels (ML) have been specified for several product categories, ranging from 0.4 mg/kg for jams, jellies, and marmalades, as well as mango chutney, to 0.01 mg/kg for infant formulae, formulae for special medical purposes intended for infants, and follow-up formulae. As for cadmium, the ML ranges from 2 mg/kg for cephalopods and marine bivalve mollusks to 0.003 mg/L for natural mineral waters. In relation to mercury in the context of the previously mentioned risks associated with fish, ML for methylmercury (the source of organic mercury for humans) ranges from 1.2 mg/kg for tuna to 1.7 mg/kg for marlin among the four fish species for which ML has been established [215]. 

The Codex Alimentarius also addresses metals essential to the healthy brain within the scope of nutrition labeling. Nutrition labeling provides label information about a food’s key nutrient content to inform consumers about the nutritional quality of the foods they purchase. The Codex Alimentarius establishes nutrient reference values (NRVs), which are a set of values used in nutrition labeling derived from authoritative recommendations for daily nutrient intake. The NRVs provided in nutrition labeling are determined based on the most current and reliable scientific knowledge regarding the daily energy or nutrient intake necessary for maintaining good health [216].

However, it is increasingly argued that the information paradigm in food-related consumer law is inefficient because it can be difficult for the majority of consumers to transform the often rather technical information on food labels into useful messages about appropriate dietary habits and a healthy lifestyle [217]. While protection against metals harmful to the brain is a relatively straightforward mechanism (food introduced to the market must meet specific criteria, which are periodically revised to align with the latest scientific advancements), implementing positive actions is more challenging, though not impossible. An excellent example of this is salt iodization. The problem of iodine deficiency in the diet, which is an essential micronutrient for the proper functioning of the human body, was addressed in the 1960s by the World Health Organization, leading to the internationalization of this problem. Currently, according to point 3.4 of the Codex Standard for Food Grade Salt, in iodine-deficient areas, food-grade salt shall be iodized to prevent iodine-deficiency disorders for public health reasons [218]. Salt iodization programs have been widely implemented across the globe to address iodine deficiency disorders and have proven to be a significant public health intervention [219]. 

Implementing a similar campaign to salt iodization for neurodegenerative diseases in the context of their association with heavy metals or essential metals is a complex matter that necessitates an in-depth understanding of the interplay between metals and diseases as well as the potential benefits and risks of such an intervention. At present, scientific evidence does not offer conclusive backing for the secure and efficient execution of such a bold initiative. Additional scientific investigations are indispensable to enhance our comprehension of the associations between metals and neurodegenerative diseases as well as their potential significance in the prevention of these conditions, particularly if such prevention is to encompass legislative changes.

## 8. Future Perspectives and Directions

Data from the current literature on trace elements and metals in neurodegenerative diseases have provided valuable insights, but these have not been merged enough overall. Several metals, including iron, copper, zinc, and aluminum, have been implicated in the pathology of these diseases. Iron accumulation, for example, is a common feature of many neurodegenerative disorders, and it has been linked to oxidative stress and neuroinflammation. However, these findings are often compartmentalized within individual disciplines, such as neurobiology, chemistry, or environmental science. Despite the progress made, there are notable deficits in the current body of research. Firstly, there is a lack of integration. The research on metals and trace elements in neurodegenerative diseases tends to occur in isolation. Interdisciplinary collaborations are still relatively rare, hindering a holistic understanding of the complex interactions involved. Encouraging greater collaboration among neuroscientists, chemists, bioinformaticians, geneticists, and environmental scientists is essential. This approach will facilitate a more comprehensive understanding of metal-related neurodegenerative mechanisms. Furthermore, it is hard to identify reliable biomarkers for metal dysregulation in neurodegenerative diseases. Such biomarkers could aid in early diagnosis and treatment monitoring. Research should focus on identifying the robust biomarkers of metal dysregulation, potentially through advanced imaging techniques, biochemical assays, and machine learning algorithms that can integrate data from various sources. Moreover, the precise mechanisms by which metals contribute to neurodegeneration remain unclear. Researchers need to delve deeper into the cellular and molecular processes involved. This involves deciphering how metals interact with proteins, affect cellular processes, and lead to neuroinflammation and oxidative stress. While there are hints at therapeutic interventions targeting metal dyshomeostasis, more work is needed to develop effective treatments or preventative strategies. This may involve the development of chelating agents, antioxidants, or lifestyle interventions to mitigate metal-related risk factors. Finally, exploring the role of environmental factors, such as pollution and dietary habits, in metal exposure and neurodegenerative risk is essential. Epidemiological studies that bridge environmental science and neurobiology can provide valuable insights. 

## 9. Summary

Trace elements and metals play crucial roles in the normal functioning of the central nervous system and are implicated in neurodegenerative diseases like Alzheimer’s disease and Parkinson’s disease. The essential trace elements zinc, copper, iron, and manganese serve as important cofactors for enzymes involved in neurotransmission, cellular metabolism, and antioxidant defense in healthy CNSs. Maintaining the proper balance of these elements is essential for neuronal integrity and cognitive function. In AD, imbalances in copper and zinc have been associated with the accumulation of amyloid-beta plaques and tau protein, contributing to cognitive decline. Similarly, the dysregulation of iron and manganese has been linked to oxidative damage and neuronal death observed in PD. Toxic metals like lead, cadmium, and aluminum have detrimental effects on CNS functioning. They impair synaptic transmission, cause neuroinflammation, and may contribute to neurodegeneration. Aluminum has also been implicated in the formation of neurofibrillary tangles in AD. Understanding the roles of these trace elements and metals in normal CNS functioning and their involvement in neurodegenerative diseases is crucial for developing potential therapeutic interventions and preventive measures. Further research is needed to fully elucidate the complex mechanisms underlying their impact on CNS health and disease, offering hope for improving the lives of individuals affected by these debilitating conditions. Utilizing legal instruments, such as the Codex Alimentarius, may play a crucial role in implementing effective actions to address these challenges and ensure public health protection in the future.

## Figures and Tables

**Table 1:** Changes in the metals in AD patients and the results they present in the brain.

Metal	General Changes	Results	References
Zinc	Extra production by nearby Zn neurons, lower levels in the brain and blood	Decreased solubility of Aβ 1-42, enhanced amyloidogenic APP cleavage, higher production of tau oligomers and clusters, tubulin aggregation, tau hyperphosphorylation, and aggregation	[86,87,88]
Copper	Elevated concentrations in plaques and tangles	Higher ability to form Aβ and its neurotoxicity	[92,93]
Iron	Buildup of iron ions in the brain,	Damage to neurons, oxidative stress in the brain,	[100,101]
	Lower iron	Easier absorption of Pb, Cd, Al, and Mn causing their buildup in the brain	[103]
Lead	Early lead exposure	Higher APP and BACE1 expression causing Aβ buildup	[106]
	Acute lead exposure	Increased Aβ buildup, higher concentration of total Tau and pTau in cells, Inflammation, microglia activation	[111,112,115,116,117]
Aluminum	Administration of Aluminum	A greater prevalence of AD or AD mortality, enhanced production of NFT, increased glial activation and inflammatory response, increased Aβ plaques and amyloid in secreted form	[119,120,128,129]
Cadmium		Contribution to forming Aβ plaques and tau aggregation	[135,137,139]

**Table 2:** Changes in the metals in PD patients and the results they present in the brain.

Metal	General Changes	Results	References
Iron	Increased total iron concentration, especially in susbtantia nigra	Neurotoxic effects	[175,176]
	Non-neuromelanin-bound iron	Possible protective effects	[177]
Manganese	Higher levels	Sensory abnormalities, neuropsychiatric and cognitive impairment, neurodegradation, manganese pathologic phenotype of PD, neurotoxicity caused by αSyn oligomerization, disruption of the cholinergic system	[172,176,177,181,184]
Mercury	Exposure	Disruption of intestinal cuprus binding causing neurotoxicity, neuroinflammation	[192,193]

## Data Availability

Not applicable.
